# Acute Diabetes-Related Complications in Patients Receiving Chemoradiotherapy for Head and Neck Cancer

**DOI:** 10.3390/curroncol31020061

**Published:** 2024-02-01

**Authors:** Rhiannon Mellor, Christian M. Girgis, Anthony Rodrigues, Charley Chen, Sonia Cuan, Parvind Gambhir, Lakmalie Perera, Michael Veness, Purnima Sundaresan, Bo Gao

**Affiliations:** 1 Crown Princess Mary Cancer Centre, Westmead Hospital, Westmead, NSW 2145, Australiamichael.veness@health.nsw.gov.au (M.V.); bo.gao@health.nsw.gov.au (B.G.); 2 Department of Diabetes and Endocrinology, Westmead Hospital, Westmead, NSW 2145, Australia; 3 Faculty of Medicine and Health, The University of Sydney School of Medicine, Camperdown, NSW 2050, Australia; 4 The Kinghorn Cancer Centre, St Vincent’s Hospital, Darlinghurst, NSW 2010, Australia; anthony.rodrigues@svha.org.au; 5 Blacktown Cancer and Haematology Centre, Blacktown Hospital, Blacktown, NSW 2148, Australia; 6 Nepean Cancer Care Centre, Nepean Hospital, Kingswood, NSW 2747, Australia

**Keywords:** head and neck cancer, chemoradiotherapy, management, diabetes, diabetes complications

## Abstract

Patients with cancer and diabetes face unique challenges. Limited data are available on diabetes management in patients undergoing concurrent chemoradiotherapy (CCRT), a curative intent anticancer therapy commonly associated with glucocorticoid administration, weight fluctuations and enteral feeds. This retrospective case–control study examined the real-world incidence of acute diabetes-related complications in patients with head and neck cancer receiving CCRT, along with the impact of diabetes on CCRT tolerance and outcomes. Methods: Consecutive patients with head and neck squamous cell or nasopharyngeal cancer who underwent definitive or adjuvant CCRT between 2010 and 2019 at two large cancer centers in Australia were included. Clinicopathological characteristics, treatment complications and outcomes were collected from medical records. Results: Of 282 patients who received CCRT, 29 (10.3%) had pre-existing type 2 diabetes. None had type 1 diabetes. The majority (74.5%) required enteral feeding. A higher proportion of patients with diabetes required admission to a high-dependency or intensive care unit (17.2 versus 4.0%, *p* = 0.003). This difference was driven by the group who required insulin at baseline (*n* = 5), of which four (80.0%) were admitted to a high-dependency unit with diabetes-related complications, and three (60.0%) required omission of at least one cycle of chemotherapy. Conclusions: Patients with diabetes requiring insulin have a high risk of acute life-threatening diabetes-related complications while receiving CCRT. We recommend multidisciplinary management involving a diabetes specialist, educator, dietitian, and pharmacist, in collaboration with the cancer care team, to better avoid these complications.

## 1. Introduction

Diabetes mellitus and cancer are commonly coexisting illnesses, and the incidence and prevalence of both are rising globally [1]. They have several common risk factors, such as aging, obesity, smoking, physical inactivity, and diet [2,3]. Diabetes itself is considered a risk factor for certain solid malignancies, including gastrointestinal and endometrial cancers [4,5]. Complications associated with diabetes such as chronic renal insufficiency, cardiovascular disease and chronic infection can also limit the use of certain anticancer therapies [5]. Finally, there is clear evidence that patients diagnosed with cancer who have pre-existing diabetes have increased postoperative mortality and poorer survival, compared to those without diabetes across all cancer types [6,7].

Definitive radiation therapy with concurrent chemotherapy for radiosensitization (CCRT) is the standard curative intent treatment for patients with locoregionally advanced head and neck squamous cell carcinoma (HNSCC), as well as locoregionally advanced nasopharyngeal carcinoma (NPC) following neoadjuvant chemotherapy. It may also be used adjuvantly in the setting of high-risk histopathological features following surgery. Cisplatin is the most commonly used chemotherapeutic agent in CCRT and has been found to induce glucose intolerance through hyperglucagonemia and impaired insulin response to hyperglycemia [8,9,10]. The use of steroids as adjunctive agents to prevent chemotherapy-induced nausea and vomiting can unmask underlying diabetes or aggravate pre-existing diabetes through reduced insulin sensitivity, increased glucose production and inhibition of the production and secretion of insulin by pancreatic beta-cells [11]. High-dose radiation therapy to the head and neck region can cause severe mucositis, odynophagia, and associated significant weight loss. Up to 74% of patients receiving CCRT for NPC or HNSCC require short-term enteral feeding [12]. These factors can substantially increase the risk of diabetes-related complications, thus potentially compromising the outcomes of anticancer treatment.

However, recommendations on management of diabetes in patients with HNSCC or NPC undergoing CCRT, particularly those requiring short-term enteral feeding, are lacking. In the context of the current scarcity of literature, we conducted a retrospective study to assess the real-world incidence of acute diabetes-related complications in patients receiving CCRT. Our secondary objective was to assess the impact of diabetes on CCRT tolerance and outcomes. Ultimately, we aim to use these data to develop a guideline for optimizing care in this population.

## 2. Materials and Methods

In this retrospective case–control study, consecutive patients who underwent definitive or adjuvant CCRT for locoregionally advanced HNSCC or NPC at two tertiary centers in Sydney Australia from 2010 to 2019 were included. To be included in the study, patients were required to be 18 years of age or older, with biopsy-confirmed squamous cell carcinoma and evidence of no distant metastatic disease. This research focused on the complications associated with the concurrent use of chemotherapy and radiation therapy; therefore, patients planned for radiation therapy alone, chemotherapy alone, or sequential chemoradiotherapy were excluded. Only patients planned for radiation therapy concurrent with cisplatin (weekly or 3 weekly) or carboplatin (weekly) chemotherapy were included. Patients who underwent CCRT with either cisplatin or carboplatin *following* neoadjuvant chemotherapy were included.

Staging was carried out according to the American Joint Committee on Cancer (AJCC) TNM 7th edition. Baseline patient demographics, tumor characteristics, disease stage, and planned therapy were collected from medical records. Outcomes of interest included treatment-related complications, diabetes-related complications, weight and feeding trends, and overall survival. Patients were classified into two groups according to whether they had diabetes at baseline or not. The group with diabetes was further divided into patients with diabetes requiring insulin at baseline and patients with diabetes not requiring insulin. Patients were classified as having diabetes if this diagnosis was listed in their background medical history. This study was approved by the Western Sydney Local Health District Human Research Ethics Committee (2010-18 QA APPROVAL).

Guidelines around initiation of enteral feeding are similar at both treatment centers. At both centers, prophylactic insertion of a percutaneous endoscopic gastrostomy (PEG) is discussed with patients prior to commencement of CCRT. If the patient consents to proceed with prophylactic PEG insertion, they are referred to the gastroenterology team for PEG insertion within +/− 14 days of CCRT initiation. Nasogastric tube (NGT) feeding is generally used where short-term enteral feeding is required following CCRT initiation and the patient does not already have a PEG (either due to complications or patient/clinician preference). The decision to start enteral feeding at both treatment centers is made by the multi-disciplinary team, in particular the Dietetics and Speech Pathology teams in discussion with the treating Radiation Oncologist. This decision is based on a combination of adequacy of oral intake and weight loss. Finally, all patients receive education from the Dietetics team, Speech Pathologist and both the Radiation Oncologist and Medical Oncologist prior to CCRT initiation. All patients are reviewed at least weekly by the Dietetics team throughout CCRT. Whilst some diabetes-specific education is included in the Dietetics review, at the time of this study, there was no mandated diabetes-specific education by a Diabetes Care Team at either treatment center.

This study was exploratory in nature, and so a formal sample size estimate was not determined. However, it was estimated that about 300 patients would meet inclusion criteria from the specified time period (2010 to 2019), of which 10–20% would have diabetes. StataBE 17 statistical software (StataCorp, College Station, TX, USA) was used for the analyses. Differences in categorical clinicopathological characteristics by diabetes status were compared using chi-square or Fisher’s exact tests, while differences in continuous variables were examined using the independent *t*-test or Mann–Whitney U test. Overall survival was estimated using the Kaplan–Meier method, and the log-rank test used to assess significance. Real-world evidence studies are at risk of bias from unmeasured confounding. Approaches to reduce the risk of bias included the use of clear, prespecified eligibility criteria and the consecutive inclusion of all patients who met inclusion criteria within the prespecified time frame. Missing data were examined, and where they were missing completely at random, complete case analysis was performed (meaning cases that had one or more values missing in any of the variables required for analysis were dropped). Where data were consistently missing from a variable across cases (for example, measured blood sugar level at specific timepoints throughout treatment), the variable itself was dropped.

## 3. Results

### 3.1. Baseline Characteristics

A total of 312 patient files were reviewed, of which 282 met criteria. Baseline characteristics according to diabetes status are displayed in Table 1. Twenty-nine (10.3%) of the patients had a preexisting diagnosis of diabetes, of which all had type 2 diabetes and five (17.2%) required insulin at baseline. Patients with diabetes were significantly older (62 vs. 56 yrs, *p* = 0.001) and were more likely to have hyperlipidemia (51.7 vs. 21.3%, *p* < 0.001), hypertension (75.9 vs. 24.1%, *p* < 0.001) and a smoking history (86.2 vs. 65.7% current or ex-smokers, *p* = 0.03). Patients with diabetes also had a significantly higher body mass index (BMI) at baseline (mean 29.7 vs. 26.9, *p* = 0.02). There was no difference between groups in terms of tumor site (*p* = 0.89) or stage (*p* = 0.34). Of the oropharyngeal carcinomas, 81 (55.5%) were related to human papilloma virus (HPV), 15 (10.3%) were HPV unrelated, and in 50 (34.2%), HPV status was unknown. This is mainly because HPV testing was not routine in the early period of this study.

Most patients in this study underwent CCRT in the definitive setting (86.5%). The most common planned chemotherapy regimen was weekly cisplatin (85.4%), followed by 3-weekly cisplatin (13.4%) and weekly carboplatin (1.2%). There was no significant difference in planned chemotherapy regimen according to diabetes status (*p* = 1.00). Forty-four patients underwent neoadjuvant chemotherapy prior to CCRT, of which the majority (88.6%) had NPC. The most common planned neoadjuvant chemotherapy regimen was cisplatin with fluorouracil (90.9%), followed by docetaxel with cisplatin and fluorouracil (TPF, 4.5%). All patients undergoing CCRT in the adjuvant setting were planned for a radiation dose of 60 Gy in 30 fractions. In the definitive setting, 161 (66.0%) were planned for a dose of 70 Gy in 35 fractions to the primary, whilst the remainder (34.0%) were planned for 66 Gy in 33 fractions. There was no difference in planned definitive radiation therapy according to diabetes status (*p* = 0.82).

### 3.2. Treatment Received, Toxicities and Complications

Fifty-eight percent of patients were able to complete their chemotherapy as planned. Fourteen patients were changed from cisplatin to carboplatin during CCRT secondary to toxicity. Mean dexamethasone dose administered with each cisplatin cycle did not differ by diabetes status (17 ± 10 mg in the group with diabetes and 17 ± 9 mg in those without diabetes, *p* = 0.86). Toxicity requiring dose reduction or omission of at least one planned cycle of chemotherapy occurred in 9 (34.6%) of the patients with diabetes and 101 (39.9%) of those without diabetes (*p* = 0.68). However, of the patients with diabetes requiring insulin at baseline, three (60.0%) had a toxicity leading to omission of at least one cycle of chemotherapy. All patients with diabetes requiring insulin at baseline were on weekly cisplatin.

Significant changes in planned radiation therapy occurred in only four (1.6%) patients, none of whom had diabetes (see Table 2). Two patients had a reduction in their total radiation dose, one patient experienced a significant treatment gap, and one experienced both a significant treatment gap and a subsequent reduction in their total radiation dose. In one case, the change in radiation therapy occurred secondary to patient preference, whilst in the other three cases, the change occurred as a result of severe toxicity.

The rate of hospitalization for toxicity was 34.5% in the group with diabetes versus 38.7% in the group without diabetes (*p* = 0.66). The most common reason for hospitalization regardless of diabetes status was secondary to the consequences of severe mucositis (45.4% of hospitalizations). However, a significantly higher proportion of patients with diabetes were admitted to a high-dependency (HDU) or intensive care unit (ICU) compared to those without diabetes (17.2% versus 4.0%, *p* = 0.003). This difference was driven largely by the subgroup of patients with diabetes requiring insulin, of which four of the five (80.0%) patients were admitted to a high-dependency unit (HDU) for a diabetes-related complication (see Table 2). Three of these admissions occurred during or within 11 days of completion of CCRT. The fourth admission occurred post-operatively, prior to adjuvant CCRT (see Table 3). The only patient with diabetes requiring insulin who was not admitted to hospital also did not require enteral feeding. None of the patients with diabetes who were on oral hypoglycemics alone at baseline had admissions for a diabetes-specific reason; however, two had admissions for other causes that were complicated by hyperglycemia requiring short-term insulin use.

### 3.3. Weight Loss and Enteral Feeds

The median time from the start of CCRT to nadir weight was 183 days (interquartile range 115 to 296 days) for the group overall. Mean weight loss was higher in the group with diabetes (15.3 versus 12.1 kg, *p* = 0.03), though when expressed as a percentage of baseline weight, this difference was no longer significant (16.6% in the group with diabetes versus 14.8% in those without diabetes, *p* = 0.20).

The majority (*n* = 210, 74.5%) of patients required enteral feeding during their treatment. There was no significant difference in the need for enteral feeds in patients without diabetes (75.8%) versus those with diabetes (67.9%), *p* = 0.36. Of those who required enteral feeding, 149 (71.0%) had their feeds administered via a percutaneous endoscopic gastrostomy (PEG), 60 (28.6%) via a nasogastric tube (NGT), and 2 (1.0%) were unknown. Enteral feeds lasted a median of 110 days (interquartile range 54 to 180). Regimens included frequent boluses of high-calorie feeds (72.4%), administration via a continuous pump (11.0%), mixed continuous/bolus regimens (9.5%), or unknown (7.6%). The most common feed type (used in 72.4%) was a 1.5 kcal/mL tube feed. When used for bolus feeding this was often administered as a 250 mL bolus (375 kcal) up to eight times a day. Rates for continuous feeds were generally 100 mLs/h (150 kcal/h).

For the patients requiring insulin at baseline, pre-mixed insulins and intermediate-acting insulins were used during continuous feeds, whilst short-acting insulin was required for bolus regimens (Table 3). Two patients had their pre-treatment insulin permanently ceased due to hypoglycemia in the context of large weight loss. Eight patients without diabetes had mildly elevated random blood glucose levels (BGLs 7.8–10.5 mmol/L) recorded during or within 12 weeks of completion of CCRT, of which five later normalized and three had no further BGLs on record.

### 3.4. Overall Survival

**Figure 1:**
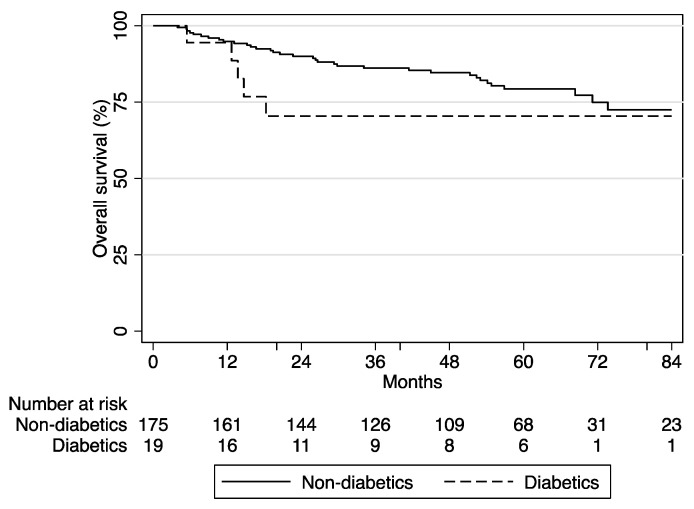
Overall survival according to diabetes status in patients undergoing definitive chemoradiation (excluding nasopharyngeal). Using the Kaplan–Meier method and log-rank test, no significant difference was found between groups, *p* = 0.08.

Survival analysis was performed in the 210 patients who underwent definitive chemoradiation, excluding those with nasopharyngeal carcinoma. The median duration of follow-up at the time of analysis was 54 months (range 4 to 132 months). The 5-year progression-free rate was 77.1% (95% CI, 70.1 to 82.7) and the 5-year survival rate was 78.4% (95% CI, 71.2 to 84.1). Of the nine patients with diabetes who died, six died due to progressive disease and three due to severe infection (two due to pneumonia and one due to a post-nasal abscess). The Kaplan–Meier curve for overall survival (OS) is shown in Figure 1. There was a non-significant trend towards poorer survival with diabetes, with a median OS of 86 months in the group with diabetes (95% CI 15, not reached) versus not reached (95% CI 127, not reached, *p* = 0.08) in the group without diabetes.

## 4. Discussion

In this retrospective cohort review, we found patients with head and neck cancer who have pre-existing type 2 diabetes requiring insulin at baseline have a high risk of severe diabetes-related complications while receiving CCRT. Whilst this represents a small total number of patients, 80% of this group required admission to a high-dependency unit and 60% required omission of at least one cycle of chemotherapy. Admissions generally occurred during CCRT or in the weeks immediately following its completion. The only patient on insulin who did not require admission for a diabetes-related complication also did not require enteral feeding. Given these findings, a protocol for diabetes management during CCRT is needed, focusing particularly on the group with diabetes who require insulin at baseline, and those requiring enteral feeding.

In keeping with previously published data, the prevalence of pre-existing diabetes in this study was 10.3% [14,15], and patients with diabetes tended to be older with more co-morbidities and a higher BMI at baseline [14,16]. There was a trend towards poorer survival with diabetes; however, cautious interpretation of this finding is required given the small numbers and mixed survival data in previous publications. Some have suggested poorer survival in HNSCC or NPC patients with diabetes [17,18], while others have suggested no significant difference [16,19].

Large weight fluctuations during CCRT and higher percentage of weight loss have been associated with poorer survival and treatment complications [20,21]. The group with diabetes in this study lost a mean 15.3 kg of weight (16.6% change from baseline) compared to 12.1 kg (14.8% change from baseline) in patients without diabetes. Time to maximal weight loss was variable but occurred a median of 183 days post CCRT completion, in line with the 6-month time to nadir weight loss quoted by Ehrsson et al. [22]. For two of the five patients with diabetes requiring insulin, this large weight fluctuation was associated with hypoglycemia and the need to eventually cease insulin.

Mucositis is almost universal in patients with head and neck cancer receiving CCRT [23] and was the most common cause of hospitalization in this study. Enteral feeding can reduce malnutrition during CCRT, reduce weight loss, and improve treatment adherence and quality of life [24,25]. Up to 74.5% of patients in this study required enteral feeding with a median duration of enteral feeds of 110 days, in keeping with the 118 days quoted in Bischoff et al. [26]. Glycemic control in patients with diabetes receiving CCRT and enteral feeds faces a multitude of challenges. Whilst crushable or dispersible oral hypoglycemics (OHG) are an option [27], administration via PEG or NGT is not recommended given the risk of tube blockage and unpredictable absorption [28]. Changes or interruptions to feeding regimens are common and may require a change in the associated anti-diabetic medication regimen. Indeed, three of the four patients who were admitted for a severe diabetes-related complication and required enteral feeding in this study also required changes in their anti-diabetic regimen. These changes involved either a switch to an insulin-only regimen (with cessation of the OHG) or change to a combination insulin with crushed OHG regimen. The only patient with diabetes requiring insulin at baseline who was not admitted for a diabetes-related complication and did not require enteral feeding was able to continue their anti-diabetic regimen throughout CCRT (which included use of an OHG). Of note, however, they did later experience a change in their anti-diabetic regimen, with cessation of insulin due to large weight loss. Weight loss of 5–7% from the side effects of CCRT can increase insulin sensitivity and require a reduction in insulin dose [29]. Finally, corticosteroids are routinely administered as antiemetics for each cycle of cisplatin chemotherapy, which can potentially trigger hyperglycemic events.

The Joint British Diabetes Societies (JBDS) released a revised guideline in 2017 for glycemic management during enteral feeding of inpatients with diabetes following a stroke [28]. The guideline emphasizes the importance of achieving a safe target glucose range without causing significant hypoglycemia, early referral to a diabetes inpatient specialist team, and capillary blood glucose monitoring every four to six hours. It makes recommendations on insulin regimens for improving glycemic control, emphasizes the importance of continuing long-acting basal insulin in patients who are on this at admission, and mentions the use of liquid metformin through the feeding tube for mild hyperglycemia in patients with type 2 diabetes. The guideline concentrates solely on control of blood glucose levels during enteral feeding in inpatients post stroke, but basic principles may be extrapolated, with the input of local diabetes teams, into other clinical situations and/or the outpatient setting.

Given up to 40% of people with diabetes may be undiagnosed in the general population [30], we recommend screening all patients with a baseline blood glucose level (BGL) or haemoglobin A1c (HbA1c) prior to CCRT. Patients with diabetes should be referred to a diabetes educator/diabetes outpatient team at the earliest opportunity, preferably prior to CCRT commencement. Their oral anti-diabetic medications or insulin regimen should be reviewed and may need to be adjusted throughout the treatment trajectory according to enteral feeding regimen used, ability to tolerate oral medications, and weight loss.

Once enteral feeding is recommended by the multidisciplinary nutritional support team, patients may need to be switched to insulin therapy, and schedules for BGL monitoring and insulin administration are likely to change [28]. Evidence to support target ranges of blood glucose in patients with diabetes receiving CCRT and enteral feeding is weak [31], with little evidence on how to best achieve this control. However, infection rate and other morbidity outcomes increase with deteriorating glucose control [32]. A fasting/pre-feed glucose range of 5–8 mmol/L and a feeding glucose range of 6–12 mmol/L have been recommended by the JBDS [28]. Patients and their family should be educated that the focus of glycemic control during CCRT should be the maintenance of blood glucose within an acceptable range while limiting the risk of hypoglycemia due to reduced oral intake from mucositis. Depending on whether feeds are continuous or intermittent/bolus, patients will need to monitor blood glucose pre-feed, 4–6 hourly during feed, and 2 h post feed. Glucose monitoring may need to be more frequent on days 1 to 4 of each cisplatin cycle, given the timing of high-dose dexamethasone administration, and may need to continue for at least 2 weeks after completion of CCRT (when the risk of complications remains high). Patients and families should be educated on what to do if feeds are stopped, and in the event of hyperglycemia or hypoglycemia.

Finally, an area of current research that may improve outcomes for all patients undergoing CCRT, particularly those with diabetes, is ‘dysphagia-optimized intensity modulated radiotherapy’ (DO-IMRT). Given the prevalence and distressing nature of dysphagia during CCRT, DO-IMRT seeks to spare organs related to swallowing, and thus reduce dysfunction [33]. DO-IMRT is associated with an improvement in patient-reported swallowing function at 12 months and beyond [33,34]. Given the complications associated with enteral feeding in patients with diabetes receiving CCRT, sparing the organs related to swallowing function in this population is a promising approach, though currently DO-IMRT has not demonstrated a significant improvement over standard IMRT in acute dysphagia or feeding tube use [33,34].

This study has its limitations. Firstly, this is a retrospective study with a small group of patients. Therefore, results may not be generalizable without larger studies. Secondly, because this study focused on the complications associated with concurrent therapy, it excluded patients who received sequential chemoradiotherapy. However, this may have excluded patients who had a planned de-escalation of therapy secondary to diabetes or other comorbidity. Finally, BGL monitoring was poorly documented in the medical records, limiting our ability to assess BGL fluctuations throughout treatment. A prospective single arm phase 2 study using a continuous glucose monitoring device is planned in patients with diabetes receiving CCRT to the head and neck or thoracic region for locally advanced head and neck cancer, lung cancer or esophageal cancer. It may provide pragmatic guidance for the outpatient management of patients receiving CCRT who require a period of enteral feeding, with the aim of improving patient outcomes and patient experience.

## 5. Conclusions

In conclusion, this study found that patients with HNSCC or NPC and type 2 diabetes requiring insulin at baseline are at high risk of life-threatening diabetes-related complications whilst undergoing CCRT, particularly if enteral feeding is initiated. The results of this study emphasize the need for a multi-disciplinary approach involving a diabetes specialist, educator, dietitian, and pharmacist in collaboration with the cancer care team. Additionally, if regular BGL monitoring is not feasible or optimal glycemic control cannot be fully achieved in the outpatient setting, inpatient treatment may be necessary in selected patients.

**Practical recommendations for patients with diabetes undergoing CCRT**

Patients with diabetes requiring insulin are at high risk of severe diabetes-related complications during and immediately following concurrent chemoradiation (CCRT). Clear guidelines are needed to prevent these complications. We recommend:

- All patients should be screened with a baseline blood glucose level (BGL) or haemoglobin A1c (HbA1c) prior to CCRT.
- Patients with diabetes should undergo close BGL monitoring during CCRT and for at least 2 weeks afterwards (when the risk of complications remains high). This is particularly important on days 1 to 4 of each cisplatin cycle, given the timing of high-dose dexamethasone
- Patients with diabetes undergoing CCRT require an integrated model of care, involving both the diabetes health care and cancer care teams. This is particularly necessary in patients requiring insulin at baseline.
- Mucositis and the requirement for enteral feeds both impact on patients’ ability to tolerate oral medications. Whilst crushable or dispersible oral hypoglycaemics are an option, administration via PEG or NGT is not recommended given the risk of tube blockage and unpredictable absorption. These patients may need to be switched to insulin therapy.
- Once enteral feeds are commenced, schedules for BGL monitoring and insulin administration change. It is therefore imperative that initiation of feeds is flagged to the wider multidisciplinary team.
- Diabetes medications may need to be adjusted throughout the treatment trajectory according to enteral feeding regimen used, ability to tolerate oral medications, and weight loss.

## Figures and Tables

**Table 1:** Baseline characteristics according to diabetes status (*n* = 282).

Characteristics	Patients with DM ^1^			Patients without DM (*n* = 253)	*p*-Value (All Patients with DM vs. Those without DM)
	Requiring Insulin (*n* = 5)	Not on Insulin (*n* = 24)	All with DM (*n* = 29)		
Male, *n* (%)	5 (100.0)	23 (95.8)	28 (96.6)	207 (81.8)	0.06
Age (mean ± SD), years	62 ± 5	62 ± 6	62 ± 5	56 ± 10	**0.001** ^3^
ECOG ^2^ 0, *n* (%)	3 (60.0)	18 (75.0)	21 (72.4)	215 (85.0)	0.77
Current/ex-smoker, *n* (%)	5 (100.0)	20 (83.3)	25 (86.2)	165 (65.7)	**0.03**
Heavy alcohol use, *n* (%)	1 (20.0)	7 (29.2)	8 (29.6)	70 (28.2)	0.88
Hyperlipidemia, *n* (%)	4 (80.0)	11 (45.8)	15 (51.7)	54 (21.3)	**<0.001**
Hypertension, *n* (%)	5 (100.0)	17 (70.8)	22 (75.9)	61 (24.1)	**<0.001**
Renal impairment, *n* (%)	3 (60.0)	4 (16.7)	7 (24.1)	86 (34.0)	0.29
BMI at baseline (mean ± SD)	29.0 ± 5.2	29.8 ± 6.6	29.7 ± 6.3	26.9 ± 5.4	**0.02**
Tumor site, *n* (%)					
Oral cavity	1 (20.0)	3 (12.5)	4 (13.8)	26 (10.3)	0.89
Nasopharynx	0 (0.0)	4 (16.7)	4 (13.8)	49 (19.4)	
Oropharynx	2 (40.0)	13 (54.2)	15 (51.7)	131 (51.8)	
Hypopharynx	1 (20.0)	3 (12.5)	4 (13.8)	28 (11.1)	
Unknown primary site	1 (20.0)	1 (4.2)	2 (6.9)	19 (7.5)	
Tumor stage, *n* (%)					
Stage II/III	1 (20.0)	13 (54.2)	14 (48.3)	99 (39.1)	0.34
Stage IV	3 (60.0)	10 (41.7)	13 (44.8)	135 (53.4)	
Unknown	1 (20.0)	1 (4.2)	2 (6.9)	19 (7.5)	
Treatment intent, *n* (%)					
Adjuvant	2 (40.0)	4 (16.7)	6 (20.7)	32 (12.7)	0.25
Definitive	3 (60.0)	20 (83.3)	23 (79.3)	221 (87.3)	
Planned concurrent chemotherapy, *n* (%)					
Weekly cisplatin (40 mg/m^2^, 6–7 cycles)	5 (100.0)	19 (79.2)	24 (82.8)	216 (85.4)	1.00
3-weekly cisplatin (100 mg/m^2^, 3 cycles)	0 (0.0)	4 (16.7)	4 (13.8)	34 (13.4)	
Weekly carboplatin (2 AUC, 6 cycles)	0 (0.0)	0 (0.0)	0 (0.0)	3 (1.2)	
Planned adjuvant radiation therapy, *n* (%) ^4^					
60 Gy in 30 fractions	2 (100.0)	4 (100.0)	6 (100.0)	32 (100.0)	-
Planned definitive radiation to primary, *n* (%) ^5^					
70 Gy in 35 fractions	2 (66.7)	14 (70.0)	16 (69.6)	147 (66.5)	0.82
66 Gy in 33 fractions	1 (33.3)	6 (30.0)	7 (30.4)	74 (33.5)	

^1^ DM = diabetes mellitus. ^2^ ECOG = Eastern Cooperative Oncology Group performance status scale, where ECOG 0 is ‘fully active, able to carry on all pre-disease performance without restriction’ [13]. ^3^ Bold values denote significance at the *p* < 0.05 level. ^4^ Expressed as a % of the total number receiving adjuvant CCRT (*n* = 38). ^5^ Expressed as a % of the total number receiving definitive CCRT (*n* = 244).

**Table 2:** Clinical outcomes according to diabetes status (*n* = 282).

Clinical Outcome	Patients with DM ^1^			Patients without DM (*n* = 253)	*p*-Value (All Patients with DM vs. those without DM)
	Requiring Insulin (*n* = 5)	Not on Insulin (*n* = 24)	All with DM (*n* = 29)		
Toxicity requiring dose reduction or omission of at least one cycle of chemotherapy, *n* (%)	3 (60.0)	6 (25.0)	9 (34.6)	101 (39.9)	0.68
Toxicity requiring reduction in radiation dose or treatment gap, *n* (%)	0 (0.0)	0 (0.0)	0 (0.0)	4 (1.6)	1.00
Toxicity requiring hospitalization, *n* (%)	4 (80.0)	8 (33.3)	12 (41.4)	98 (38.7)	0.78
Admission to HDU ^2^ or ICU ^3^, *n* (%)	4 (80.0)	1 (4.2)	5 (17.2)	10 (4.0)	**0.003** ^4^
Total weight lost (mean ± SD), kg	13.8 ± 7.9	15.6 ± 9.7	15.3 ± 9.3	12.1 ± 6.8	**0.03**
Time to maximum weight lost (median, IQR), days	126 (113–148)	147 (117–272)	146 (113–252)	188 (116–303)	0.29

^1^ DM = diabetes mellitus. ^2^ HDU = high-dependency unit. ^3^ ICU = intensive care unit. ^4^ Bold values denote significance at the *p* < 0.05 level.

**Table 3:** Patients with type 2 diabetes requiring insulin at baseline (*n* = 5): enteral feeding regimens and diabetes-related complications.

Diabetes Complication	Timing of Admission	Baseline Diabetes Medications	Enteral Feeding Regimen	Diabetes Medications during Feeds (Frequency)	Diabetes Medications Post Feed Cessation
Admitted with high risk of HHS ^1^	Post-operatively, pre-CCRT ^2^	Metformin Dapagliflozin Gliclazide Insulin glargine	Continuous	Isophane insulin (BD ^3^ or TDS ^4^), insulin human (TDS or QID ^5^)	Metformin Gliclazide Insulin glargine Insulin aspart
Admitted with HHS	11 days post CCRT	Metformin Insulin glargine Insulin aspart	Initially continuous	Insulin glargine (BD), insulin aspart (PRN ^6^).	Insulin aspart pre-feeds, metformin. Insulin glargine not restarted due to weight loss
			Later bolus	Insulin aspart (pre-feeds), metformin (crushed).	
Admitted with DKA ^7^	10 days post CCRT	Metformin Insulin glargine Insulin aspart	Nil	Not applicable	Not applicable
Admitted with DKWA ^8^	Day 34 of CCRT	Insulin aspart/protamine Insulin aspart	Initially continuous	Insulin aspart/protamine, insulin aspart (BD)	Insulin aspart/protamine Insulin aspart
			Later bolus	Insulin glargine (OD ^9^), insulin human (every 2nd feed)	
Nil	Not applicable	Insulin aspart Gliclazide	Nil	Not applicable	Gliclazide. Insulin aspart ceased due to weight loss

^1^ HHS = hyperosmolar hyperglycemic syndrome. ^2^ CCRT = concurrent chemoradiotherapy. ^3^ BD = twice daily. ^4^ TDS = three times a day. ^5^ QID = four times a day. ^6^ PRN = as required. ^7^ DKA = diabetic ketoacidosis. ^8^ DKWA = diabetic ketosis without acidosis. ^9^ OD = daily.

## Data Availability

Data are encrypted and are available on reasonable request from the authors.
